# Attention to Local Health Burden and the Global Disparity of Health Research

**DOI:** 10.1371/journal.pone.0090147

**Published:** 2014-04-01

**Authors:** James A. Evans, Jae-Mahn Shim, John P. A. Ioannidis

**Affiliations:** 1 Department of Sociology, Computation Institute and Center for Health and the Social Sciences, University of Chicago, Chicago, Illinois, United States of America; 2 Department of Sociology, University of Seoul, Seoul, Korea; 3 Departments of Medicine, Health Research and Policy, and Statistics, Stanford Prevention Research Center, Stanford University, Stanford, California, United States of America; Toronto Western Hospital, Canada

## Abstract

Most studies on global health inequality consider unequal health care and socio-economic conditions but neglect inequality in the production of health knowledge relevant to addressing disease burden. We demonstrate this inequality and identify likely causes. Using disability-adjusted life years (DALYs) for 111 prominent medical conditions, assessed globally and nationally by the World Health Organization, we linked DALYs with MEDLINE articles for each condition to assess the influence of DALY-based global disease burden, compared to the global market for treatment, on the production of relevant MEDLINE articles, systematic reviews, clinical trials and research using animal models vs. humans. We then explored how DALYs, wealth, and the production of research within countries correlate with this global pattern. We show that global DALYs for each condition had a small, significant *negative* relationship with the production of each type of MEDLINE articles for that condition. Local processes of health research appear to be behind this. Clinical trials and animal studies but not systematic reviews produced within countries were strongly guided by local DALYs. More and less developed countries had very different disease profiles and rich countries publish much more than poor countries. Accordingly, conditions common to developed countries garnered more clinical research than those common to less developed countries. Many of the health needs in less developed countries do not attract attention among developed country researchers who produce the vast majority of global health knowledge—including clinical trials—in response to their own local needs. This raises concern about the amount of knowledge relevant to poor populations deficient in their own research infrastructure. We recommend measures to address this critical dimension of global health inequality.

## Introduction

Poor and minority persons, as well as those living in resource restricted regions, are more likely to live shorter, less healthy lives [1], [2], [3]. A long tradition in medicine has sought to reduce these inequities and realize universal, global health through socio-economic development, improved public health measures and affordable health care [4], [5], [6], [7]. Despite successes, there remain concerns about the relevance and effectiveness of these efforts for disadvantaged populations. Target populations are sometimes resistant and non-adherent to medical intervention. This has inspired educational projects to enhance the public understanding of medicine [8], [9] and practitioner understanding of diverse patient cultures.

Doubts persist, however, about whether we produce sufficient medical knowledge to provide medical care for certain conditions in certain contexts [10], [11], [12], [13]. Counter-intuitive findings about emergency care for African children suffering from malaria, septicemia, meningitis and similar infectious diseases suggest that we know much less about diagnosis and treatment for poor populations [14], [15], [16]. Moreover, effective therapies for pandemics such as HIV can create unforeseen knowledge needs like how to provide long-term medical care among HIV survivors [17], [18].

Here we examine whether the global research community has given sufficient attention to medical conditions prevailing in globally disadvantaged populations. We demonstrate how this concern follows from the misalignment of global disease burden and global research attention. Specifically, we reveal the global inequality of health research by estimating the relationship between the health burden imposed by many important diseases and subsequent publication of biomedical articles relevant to those diseases. We also explore possible causes for this inequality of health research.

Our findings highlight how poor populations not only face the greatest burden from disease and disability, but that burden is given the least medical research attention. We show that this global inequality of health research follows from two processes. First, medical research activities are guided by local health needs specific to each country rather than global health needs, and health needs vary greatly across rich and poor populations. Second, as medical research requires resources, a few developed countries disproportionately produce the vast majority of biomedical research. As a result, global research attention to diseases tracks the global market for treatment and the ability of patients to pay for care. This has resulted in the current global inequality of health research. To reduce this inequality of research, our analysis recommends efforts that not only globalize the research attention of wealthy countries [19], but also support local research in those impoverished contexts where health knowledge is needed most.

## Materials and Methods

### Study Design and Key Measures

We assessed the total number of biomedical articles and also the specific number of systematic reviews, randomized controlled trials and animal research relevant to a wide range of specific diseases and disabilities, and then explored how much of these distributions could be explained by 1) the global health burden imposed by these conditions, 2) the global market for medical treatment, and 3) the local health burden. We investigated these relationships further by assessing differences in the health profiles of developed and less developed countries, and by measuring the association between a country's GDP and its production of biomedical science.

To measure the amount of disease-specific biomedical research, we used *the total number of articles* published on each disease in MEDLINE. We also calculated the precise number of *systematic reviews*, *randomized controlled trials*, and research performed on *animal subjects* devoted to those same conditions. Each abstract in MEDLINE is indexed with NLM Medical Subject Headings (MeSH) [20], [21]. We defined MEDLINE papers as relevant to one or more diseases if annotated with related MeSH clinical and disease terms. We assessed this for each country by linking MEDLINE with Thomson Reuters' Web of Science, which provides full institutional information for most MEDLINE articles. We then coded the countries of the institutions that hosted each article author. See File S1 for details.

Number of total research articles is a reasonable indicator of health research, but an imperfect proxy for biomedical knowledge more generally: some diseases are harder to understand, prevent, diagnose, and treat than others. For this reason, we also assessed the number of different types of articles: systematic reviews, randomized controlled clinical trials, and animal subjects research associated with each disease. The number of systematic reviews indicates that the biomedical community deems research on a disease of sufficient size, and relevance that it merits secondary evaluation and organization. The number of clinical trials is a marker of organized research assessing the merits of interventions for a condition across many patients in one or multiple centers. Finally, the number of research papers performed on animals suggests an interest at fundamental aspects of each disease.

It should be noted that medical science can possess deep knowledge of a disease that continues to cause harm because that knowledge has not yet disseminated to places where it is needed most. Nevertheless, recent studies that demonstrate our limited knowledge about treatment in resource poor environments [22] suggest that even for diseases about which we have extensive biological understanding, additional research into their distribution, acquisition, prevention and treatment among different populations and in different contexts would likely produce further, much needed medical insight. Following this, we believe that number of total articles provides a useful purchase on relevant health knowledge as those articles cover the range of health research, taking biological but also behavioral, social, economic, political and cultural factors into account, as many do here. Number of systematic reviews, randomized control clinical trials, and disease-relevant research performed on animal models provide more fine-grained insight about the relationship between health burden, research and treatment.

We used World Health Organization (WHO) data to measure *the burden of disease*. The WHO introduced global and regional, but not country-level, estimates of the disability-adjusted life years (DALYs) for an array of common conditions through its Global Burden of Disease (GBD) project in 1990 [23]. In 2002 and 2004, the WHO re-estimated DALYs for 192 countries as well as globally [24]. One DALY refers to one healthy life year lost to disease or disability. By converting time spent in various states of health to their “healthy-year equivalents,” [25] DALYs incorporate cultural values placed on different aspects of physical, mental and social function [26]. The WHO estimates DALYs for 136 health conditions. We used 111 of the 136 conditions in our analysis, excluding residual categories like “other infectious diseases.” GBD codes for these conditions are organized into 19 categories, and 3 high-level classifications (see Table S1 in File S1 for all codes).

We matched GBD codes to MeSH terms through the mediation of ICD-9 (International Statistical Classification of Diseases and Related Health Problems) codes. ICD-9 codes are sufficiently general that we mapped them onto GBD codes with very little ambiguity. We then linked ICD-9 codes to MeSH through NLM's Unified Medical Language System (UMLS) metathesaurus. Following this approach, we regrouped MeSH disease terms according to the 111 GBD codes and so estimated the number of articles in MEDLINE relevant to a particular disease category (see File S1 for alternate linkages).

We measured *the global market for treatment* associated with each disease. First, we multiplied the number of disability-adjusted life years (DALYs) for each disease in each country by gross national income per capita (GNI) at purchasing power parity (PPP) in that country. This product equals the value of the revenue that could be generated if everyone afflicted by the condition in question was restored to full health, or the size of a national market for treatment, if people in that country were willing to spend all money that could be gained from health on health. With the same disease profile, different countries have different markets, depending on their GNI. We used the World Bank's World Development Indicators for GNI (PPP) data for each country [27]. By summing all national markets for treatment for a given condition, we computed the global market for treatment for that condition. The global market for a condition common in developed countries is much greater than the market for a condition prevalent only among less developed countries.

### Statistical Analysis

We used regression-based analyses to estimate the association between the burden of disease and the market for treatment on the quantity of medical research produced. Counts for each type of disease-relevant article are not normally distributed: they are discrete and widely skewed with a few diseases like breast cancer and AIDS attracting a disproportionate share of research attention while others like Chagas disease and leishmaniasis attracting little [19]. This recommended the use of negative binomial regression models.

First, we analyzed the relationship between the global burden of disease for 111 diseases and disabilities in one year (2002 and 2004) and the global number of articles published relevant to those conditions in the subsequent year. We subsequently analyzed the relationship of the market for treatment on the quantity of subsequently published science. This analysis involved 4,703,021 disease and disability assignments to 3,771,604 distinct articles.

Our next analysis evaluated the correlation of burden of disease within countries on the number of subsequently published medical articles, systematic reviews, randomized-controlled clinical trials, and animal model studies relevant to each disease by researchers from those countries. We estimated these models with data from the 167 countries for which total article information was complete and 155 countries for which information about systematic reviews, randomized-controlled clinical trials and animal models studies was complete.

Finally, we explored the relationship between poverty, disease and disease-relevant science revealed by our regression analyses. We examined the difference in disease distribution between developed and less developed countries by calculating the relative burden of disease for each country and then graphing and modeling its relationship with that country's gross domestic product (GDP). We also assessed the relationship between wealth and the amount of research published by researchers in each country.

## Results

**Figure 1:**
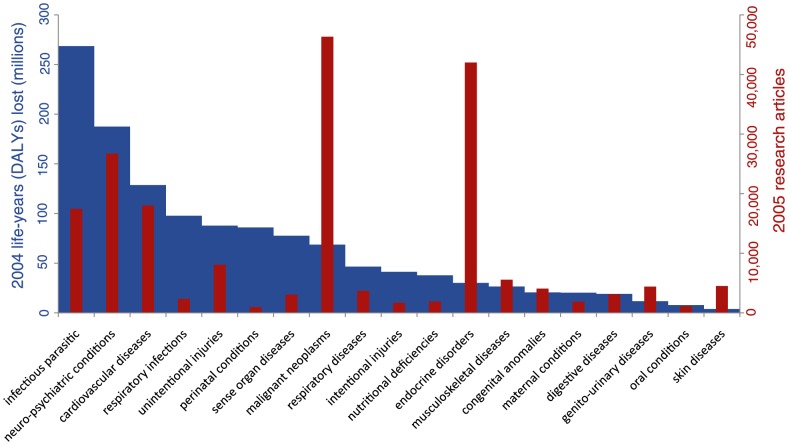
2004 global disability-adjusted life years (DALYs) and 2005 research articles categorized by 19 broad WHO disease and disability categories. This correspondence suggests the loose relationship between burden of disease and health knowledge (see Figure S1 in File S1 for the distribution of different types of articles by disease).

Our world-level analysis reveals that the global burden of disease accounts for *none* of the distribution of total health research or the controlled trials published in the subsequent year (see Table 1). For randomized controlled trials and animal model studies, more global need is actually associated with less global research. Systematic reviews responds positively to global DALYs, but only when the 19 broad disease and disability categories are statistically controlled for. Figure 1 summarizes the alignment of health burden with health research, as grouped by broad disease and disability category. This illustrates how malignant neoplasms (cancers), endocrine disorders (including diabetes), and skin diseases are overrepresented in biomedical research, disproportionate to the global health burden they exact. In contrast, infectious parasitic disorders, respiratory infections and perinatal conditions are underrepresented in the research relative to their burden.

**Table 1:** Estimated Association of Global Biomedical Articles with Global Health Burden (2002, 2004)^a^.

*Model 1: All research articles*	% change	95% C.I.	% change	95% C.I.	% change	95% C.I.
**Global DALYs (10 millions)**	−5.1	−10.7–1.0	−3.9	−9.3–1.7	3.3	−3.1–10.2
**Market size ($10 billions)**	3.6**	2.2–5.0	3.0**	1.6–4.4	1.3†	−0.1–2.8
**Cumulative Global articles (10 thousands)**			3.0**	2.2–3.8	10.3**	7.0–13.6
*Model 2: Systematic reviews*						
**Global DALYs (10 millions)**	−0.9	−10.3–9.4	0.8	−8.6–10.2	11.6†	−1.8–25.1
**Market size ($10 billions)**	2.7**	0.8–4.6	2.0*	0.1–3.9	0.4	−1.8–2.7
**Cumulative Global articles (10 thousands)**			23.5**	14.0–33.8	12.5**	5.5–19.9
*Model 3: Clinical trials*						
**Global DALYs (10 millions)**	−11.2*	−20.9–−0.3	−7.1	−13.1–8.1	6.6	−6.0–20.9
**Market size ($10 billions)**	5.2**	2.5–7.9	3.2*	−1.6–3.0	1.1	−1.5–3.8
**Cumulative Global articles (10 thousands)**			561.8**	275.8–1065.7	341.2**	142.2–703.6
*Model 4: Animal subjects*						
**Global DALYs (10 millions)**	−8.0†	−15.8–0.5	−4.7	−14.0–3.6	1.1	−7.4–10.2
**Market size ($10 billions)**	3.8**	1.1–6.5	1.9†	−0.4–3.0	1.0	−1.3–3.3
**Cumulative Global articles (10 thousands)**			35.9**	26.7–45.8	29.1**	19.1–39.9
					Controlling for disease‡	

a Models in 1A contain 222 cases (111 diseases in 2002 and 2004).

**Figure 2:**
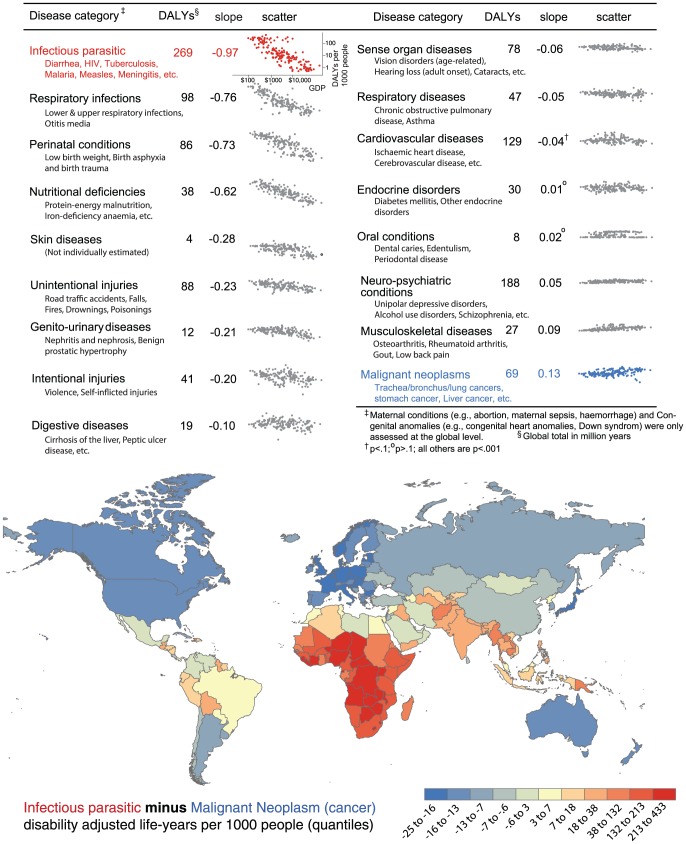
Broad disease categories, the global DALYs they exact, and the relationship between country health burden and wealth for broad disease categories. Disease subcategories (e.g., HIV/AIDS) are listed in order from those that incur the largest global health burden. Scatter plots graph country DALY rate (DALYs per 1000 people) of conditions by GDP per capita, plotted on a log scale; slopes represent this as a linear relationship (the estimated OLS coefficient of logged GDP per capita regressed on logged DALY rate). The global map illustrates country differences in disease burden by plotting the difference between DALY rate for infectious diseases and cancers, categories with the most negative and positive relationship with country wealth.

‡ Models control for the 19 broad disease/disability categories listed in Figure 2 (including the 2 in the footnote).

† *p* <.10;

* *p* <.05;

** *p* <.01

In contrast, the global market for treatment significantly impacts health research. Table 1 shows that for every $10 billion lost to a disease or disability, which might have been put toward care, biomedical articles of all types of controlled trials devoted to that disease increased by approximately 3–5% in the subsequent year, controlling for health burden. Randomized control trials, which are most expensive and closest to marketable health products, increase most—by 5.2% the following year. These statistical relationships between the number of articles of various types, the burden of disease, and the global market for treatment persist when we controlled for the total cumulative number of articles relevant to each condition and the proportion of those articles published in the prior five years, but they attenuate when we include indicator variables for 19 coarse disease and disability categories. This means that much of the positive effect of market size on published research is attributable to different categories of disease, which are associated with larger and smaller markets. These patterns remained unchanged in a supplementary analysis using global DALY data from 1990 and 2004 (eTable 2). In this analysis, disease burden remains insignificant, but growth in market size (by $10 billion) leads to an increase of relevant articles by more than 10%, which attenuates when controlling for disease categories. These results together show that diseases prevailing in poor populations are given less overall research attention than those common in wealthy populations. Although market forces appear to be implicated in the publication of research articles of all types, in the following analyses we show how they are likely not the root cause of unequal health knowledge, but themselves a consequence of global health and wealth inequality.

**Table 2:** Estimated Association of National Biomedical Articles with National Health Burden (2002, 2004)^a^.

*Model 1: All research articles*	% change	95% C.I.	% change	95% C.I.	% change	95% C.I.
**National DALYs (10 millions)**	73.9**	17.4–130.5	72.4**	18.0–126.7	68.4**	15.1–121.7
**Global DALYs (10 millions)**	1.0*	−0.9–2.8	0.7**	−1.1–2.5	5.0**	3.0–7.0
**Cumulative National articles (thousands)**			1.1**	0.8–1.3	1.2**	1.0–1.4
**Cumulative Global articles (thousands)**			0.3**	0.2–0.3	0.3**	0.2–0.3
*Model 2: Systematic reviews*						
**National DALYs (10 millions)**	27.4	−41.6–96.4	23.9	−41.4–89.2	23.5	−41.7–88.7
**Global DALYs (10 millions)**	0.2	−3.0–3.4	−0.1	−3.2–3.0	7.5**	3.9–11.1
**Cumulative National articles (thousands)**			7.3**	5.0–9.5	8.9**	6.6–11.2
**Cumulative Global articles (thousands)**			2.5**	2.0–2.9	1.7**	1.1–2.3
*Model 3: Clinical trials*						
**National DALYs (10 millions)**	367.9**	92.3–1038.6	297.6**	80.8–774.6	285.6**	81.7–718.3
**Global DALYs (10 millions)**	3.1†	−0.1–6.3	1.9	−1.1–4.9	7.9**	4.5–11.5
**Cumulative National articles (thousands)**			57.7**	41.7–75.5	73.9**	56.0–93.8
**Cumulative Global articles (thousands)**			26.9**	23.2–30.7	16.9**	11.9–22.0
*Model 4: Animal subjects*						
**National DALYs (10 millions)**	90.8**	−42.5–90.4	87.1**	20.3–190.9	81.1**	17.0–180.3
**Global DALYs (10 millions)**	0.6	−2.9–3.4	0.4	−1.8–2.6	1.4	−1.1–3.9
**Cumulative National articles (thousands)**			10.7**	9.3–12.2	11.1**	9.6–12.5
**Cumulative Global articles (thousands)**			1.4**	1.1–1.7	1.2**	0.7–1.6
					Controlling for disease‡	

a Models in 2B contain 8102 cases (up to 111 diseases and 192 countries in 2002 and/or 2004).

‡ Models control for the 19 broad disease/disability categories listed in Figure 2 (including the 2 in the footnote).

† *p*<.10;

* *p*<.05;

** *p*<.01.

Our next analysis shows that within countries, disease burden has a strong, significant association with many forms of health research. For each 10 million DALYs lost to a disease within a country, the number of articles published by researchers in that country increased by 73.9% (see Table 2). The effect of local burden is highest for randomized controlled clinical trials, where a million DALYs lost to a disease results in 367.9% more such trials in that year. Only the number of systematic reviews on a disease do not vary significantly with recent DALYs lost to that disease within country. Interestingly, systematic reviews do not respond more to the amount of previous national or global research than other kinds of research.

Global burden of disease has a small, independent association with the publication of all research articles within countries, and with review articles and all clinical research when controlling for broad disease categories. This suggests that whether or not researchers and funding agencies factor global health needs into their research, the influence of local needs exerts much more influence on their work. Alternative specifications of country-authorship produced the same pattern of results (i.e., all countries with participating authors are assigned the article versus only the wealthiest country, which restricts the measure to indigenous research; see File S1.

In order to reconcile the presence of a national association between health burden and health research with the absence of a global one, we explored how disease profiles and health research are correlated with national wealth.

Figure 2 illustrates the striking difference in disease profiles among populations of rich and poor countries. These are evident at the level of coarse disease classifications, but the differences are much larger at the level of individual conditions (see Table S3 and Figure S1 in File S1). For example, consider the relative burden of infectious and malignant neoplasms (cancers) in rich and poor countries. Infectious diseases like diarrheal diseases, malaria and HIV naturally levy a much higher toll in less developed countries, while cancers incur a larger burden in more developed countries with longer life spans. Respiratory infections, perinatal conditions and injuries disproportionately afflict less developed countries, while neuro-psychiatric conditions like depression and schizophrenia and musculoskeletal diseases like arthritis and back pain represent a greater burden in wealthy countries. Note the conditions that most afflict poor populations only lightly affect the rich (e.g., infectious diseases, respiratory infections, perinatal conditions), while diseases that most afflict rich populations also levy a substantial toll on poor ones (e.g., cancers, neuro-psychiatric and musculoskeletal disorders). Figure 2 also shows the regional dispersion of these health burden differences. The world's least developed countries are located in Africa, and to a lesser extent South Asia and South America: so also disease burden clusters regionally, largely correlated with country wealth.

**Figure 3:**
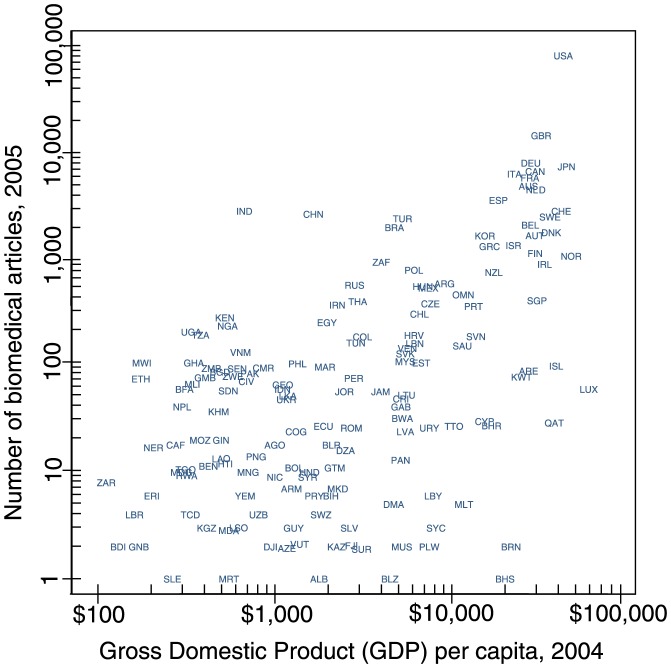
Relationship between the national GDP per capita in 2004 and the quantity of research published by researchers in 2005, by country, plotted on a logarithmic scale (to spread out countries for visual inspection). Each three character string corresponds to the unique ISO 3166-1 alpha-3 code associated with each country (see Table S4 in File S1 for complete list).

There are, however, striking disparities among countries in the capacity to produce health research. Figure 3 plots the relationship between country wealth and the publication of biomedical research. This figure illustrates how wealthy countries publish much more biomedical research than less wealthy countries. National disparities in research are not surprising, as biomedical research requires substantial resources. Nevertheless, combined with the responsiveness to local health needs demonstrated previously, research disparities result in the overrepresentation of conditions burdening developed countries and the underrepresentation of those afflicting less developed countries in the research literature. The inequality of research limits current quality of care in less developed countries, but it also limits the next generation of care there, as the science and technology that could be transferred to developing countries are less relevant to their most pressing health needs.

## Discussion

Our analysis demonstrates that the production of health research in the world correlates with the market for treatment and not the burden of disease. While we expected to find a weak relationship between global health and medical research, we find *no relationship*. One prior study that found a modest correlation between the amount of systematic review papers and global burden of disease [11], our research reveals the fragile nature of this relationship. Clinical trails and case reports have no relationship to global burden, and systematic reviews only post a small influence when disease categories are held constant. This means that existing global health research is less relevant to the needs of poor populations.

More importantly, we show how this global pattern is related to the local processes of health research: 1) local health needs within a country draw the attention of researchers and research resources of the country more than global health needs [13], [19], 2) developed countries and less developed countries have divergent health profiles, and 3) developed countries produce much more health research of all kinds than less developed countries [10]. In short, health needs from less developed countries do not attract much attention among rich country researchers. Ultimately, this article stresses that poor populations are in double jeopardy: they experience the greatest health burdens but their diseases have been studied least and even researchers from wealthy countries often lack secure knowledge for context-relevant treatments. Systematic reviews, which are not driven by local needs, attend to research slightly more relevant to global health needs, but this correction is very small.

These findings have relevance for international development and health policy. The primary focus of international health efforts has been to extend health care innovations from developed countries to less developed and comparatively less healthy countries. This is good policy: as we demonstrate, conditions that incur the highest health burden in wealthy countries like cancers and musculoskeletal disorders are relevant also to poor countries. They are, however, not the most burdensome health challenges for those countries. Even global health initiatives, which often target specific diseases relevant to less developed countries and have succeeded in reducing some health inequities, are not always sufficiently aligned with country priorities or countries' burdens of disease [28], [29].

Others argue that the biggest health challenges are the result of inferior environmental contexts (e.g., increased air and water pollution or sanitation), and so efforts to reduce global health inequity should focus on economic development and public health. This position is not unreasonable, but understates the possibility that we lack appropriate knowledge to intervene in impoverished environments or those simply different from rich countries. For example, recent research demonstrates that child hydration, a long-promoted emergency care measure for children suffering from infectious disease in resource poor sub-Saharan Africa increases short-term mortality [22]. One conclusion voiced by Ugandan doctor Peter Oluput-Oluput is that “we need to do more research in Africa for Africans.” [30] This suggests the importance of transferring not only health technology like tertiary care facilities to the least developed countries, but also helping to transfer *health research technology* to impoverished locations with health burdens that differ most from wealthy countries. These recommendations do not presume what less developed countries should want or how they should spend their limited resources and balance urgent with long-term health needs. Neither do they address the material inequalities that lie behind inequality in both health and health knowledge. Our research simply highlights the potential impact of more health research relevant to the needs of the poorest populations.

Not only environmental but the biological context of disease is likely to be different in less developed countries. Research suggesting that treatment for some cancers may be less effective in certain U.S. minority populations suggests that current therapies may have been “overfit” to a biased sample of genes and bodies [31]. A growing collection of related findings have been framed as evidence that biological factors play a role in health disparities [32], [33], [34], [35], [36], but they also implicate the differential relevance of health knowledge produced by biomedical research for the health of different groups [37]. In short, the same care may not always be equal. In this way, the inequality of biomedical research that our analysis demonstrates likely understates its true inequality.

By estimating the particular inequality of health conditions in relation to national wealth (see Figure S1 in File S1), our study highlights those most likely to be underserved given the national focus and global inequality of research funding. For example, malaria, tetanus, Chagas disease, measles, Vitamin A deficiency, lymphatic filariasis, schistosomiasis, and diphtheria most disproportionately afflict poor populations. Other conditions also inflict a greater burden in less developed countries, including fires, violence, drowning, and poisoning, as also glaucoma, peptic ulcers and ear infections.

Our study has several limitations. The national-level burden of disease data are only for two years, two years apart, which does not provide sufficient change to isolate a causal effect of health burden on research (see File S1). The lag between burden of disease and disease-relevant publication may also not be long enough to demonstrate the total influence—we had only one year of subsequent citation data available to us. Moreover, we did not have data on the economic value of diseases within countries, and so we were unable to explore to what degree the same dynamic that occurs across countries occurs inside them. Finally, we neglect several other institutions that likely influence health research, independent of global health needs. These include national funding priorities, activism in disease communities, the scientific maturity or generality of research on some disorders over others, etc. Nevertheless, we believe that our analysis sheds light on the global inequality of health research and suggests that attention to local disease is likely a primary influence. To address global health inequality, we propose the development of measures to globalize the research attention of wealthy countries and to support local research in impoverished contexts where health knowledge is needed most.

## Supporting Information

File S1 **Supporting information, figures, and tables.** **Figure S1, 2004 global disability-adjusted life years (DALYs) and 2005 reviews, clinical trials and animal studies categorized by 19 broad WHO disease and disability categories.** This correspondence the loose relationship between burden of disease and health knowledge (see Figure 1). **Figure S2, Relationship between national disease burden and wealth.** Scatterplots of national DALY rate (DALYs per 1000 people) and GNI per capita for each of 96 specific health conditions, where each point is a country. Also shown is the estimated influence (or regression slope) of logged DALY rate on logged GNI per capita, by condition, computed using ordinary least-squares (OLS) regression. **Figure S3, Relationship between the national GDP per capita in 2004 and the quantity of reviews, clinical trials and animal studies published by researchers in 2005, by country, plotted on a logarithmic scale (to spread out countries for visual inspection).** Each three character string corresponds to the unique ISO 3166-1 alpha-3 code associated with each country (see Figure 3 and Table S4 in File S1 for complete list). **Table S1, Complete list of WHO Global Burden of Disease Categories. Table S2, Estimated Change in Global Number of Biomedical Articles with Changes in Global Health Burden (1990, 2004). Table S3, Estimated Change in Regional Number of Biomedical Articles with Changes in Regional Health Burden (1990, 2004). Table S4, Disease or Disease Category exacting the most DALYs. Table S5, Countries and their 3-Character Codes from** **Figure 3** **.**
(DOCX)

## References

[1] Henschke UK, Leffall LD Jr, Mason CH, Reinhold AW, Schneider RL, et al (1973) Alarming increase of the cancer mortality in the U.S. black population (1950–1967). Cancer 31: 763–768. 4706044 10.1002/1097-0142(197304)31:4<763::aid-cncr2820310401>3.0.co;2-s

[2] Medicine Io (2003) Unequal Treatment: Confronting Racial and Ethnic Disparities in Health care. Washington, DC: National Academy of Sciences.

[3] Marmot M (2005) Social determinants of health inequalities. Lancet 365: 1099–1104. 15781105 10.1016/S0140-6736(05)71146-6

[4] Lurie N (2005) Health disparities–less talk, more action. The New England Journal of Medicine 353: 727–729. 16107626 10.1056/NEJMe058143

[5] Vaccarino V, Rathore SS, Wenger NK, Frederick PD, Abramson JL, et al (2005) Sex and racial differences in the management of acute myocardial infarction, 1994 through 2002. The New England Journal of Medicine 353: 671–682. 16107620 10.1056/NEJMsa032214 PMC2805130

[6] Jha AK, Fisher ES, Li Z, Orav EJ, Epstein AM (2005) Racial trends in the use of major procedures among the elderly. The New England Journal of Medicine 353: 683–691. 16107621 10.1056/NEJMsa050672

[7] Trivedi AN, Zaslavsky AM, Schneider EC, Ayanian JZ (2005) Trends in the quality of care and racial disparities in Medicare managed care. The New England Journal of Medicine 353: 692–700. 16107622 10.1056/NEJMsa051207

[8] Aizer A, Stroud L (2010) Education, Knowledge and the Evolution of Disparities in Health. National Bureau of Economic Research Working Paper Series No 15840.

[9] Stephenson J (2002) AIDS Knowledge Gap. JAMA: The Journal of the American Medical Association 288: 155–155. 12095370

[10] International Working Party to Promote and Revitalise Academic Medicine (2004) Academic Medicine: The Evidence Base. International Working Party To Promote And Revitalise Academic Medicine. BMJ: British Medical Journal 329: 789–792. 15459055 10.1136/bmj.329.7469.789 PMC521006

[11] Swingler GH, Volmink J, Ioannidis JPA (2003) Number of Published Systematic Reviews and Global Burden of Disease: Database Analysis. BMJ: British Medical Journal 327: 1083–1084. 14604930 10.1136/bmj.327.7423.1083 PMC261743

[12] Swingler GH, Pillay V, Pienaar ED, Ioannidis JPA (2005) International Collaboration, Funding and Association with Burden of Disease in Randomized Controlled Trials in Africa. Bulletin of the World Health Organization 83: 511–517. 16175825 PMC2626288

[13] Isaakidis P, Swingler GH, Pienaar ED, Volmink J, Ioannidis JPA (2002) Relation between Burden of Disease and Randomised Evidence in sub-Saharan Africa: Survey of Research. BMJ: British Medical Journal 324: 1–5. 11909786 10.1136/bmj.324.7339.702 PMC99053

[14] RTSS CTP (2011) First Results of Phase 3 Trial of RTS,S/AS01 Malaria Vaccine in African Children. New England Journal of Medicine 10.1056/NEJMoa1102287 22007715

[15] Boseley S (2011) Malaria Vaccine Set to Save Millions of Lives, But Who Will Fund It? The Guardian

[16] Maitland K, Kiguli S, Opoka RO, Engoru C, Olupot-Olupot P, et al (2011) Mortality after Fluid Bolus in African Children with Severe Infection. New England Journal of Medicine 364: 2483–2495. 21615299 10.1056/NEJMoa1101549

[17] Buscher AL, Giordano TP (2010) Gaps in Knowledge in Caring for HIV Survivors Long-term. JAMA: The Journal of the American Medical Association 304: 340–341. 20639568 10.1001/jama.2010.870

[18] Holmboe ES, Lipner R, Greiner A (2008) Assessing Quality of Care. JAMA: The Journal of the American Medical Association 299: 338–340. 18212320 10.1001/jama.299.3.338

[19] Bartlett C, Sterne J, Egger M (2002) What is newsworthy? Longitudinal study of the reporting of medical research in two British newspapers. BMJ 325: 81–84. 12114239 10.1136/bmj.325.7355.81 PMC117129

[20] Lipscomb C (2000) Medical Subject Headings (MeSH). Bulletin of the Medical Library Association 88: 265–266. 10928714 PMC35238

[21] Rogers FB (2000) Medical subject headings. Bulletin of the Medical Library Association 88: 114–116. PMC197951 13982385

[22] Maitland K, Kiguli S, Opoka RO, Engoru C, Olupot-Olupot P, et al (2011) Mortality after Fluid Bolus in African Children with Severe Infection. The New England Journal of Medicine May 26. 10.1056/NEJMoa1101549 21615299

[23] Murray CJL, Lopez AD (1996) The global burden of disease : a comprehensive assessment of mortality and disability from diseases, injuries, and risk factors in 1990 and projected to 2020. Cambridge, MA: Published by the Harvard School of Public Health on behalf of the World Health Organization and the World Bank ;Distributed by Harvard University Press. xxxii, 990 p.p.

[24] Mathers C, Fat DM, Boerma JT (2008) The global burden of disease : 2004 update. Geneva, Switzerland: World Health Organization. vii, 146 p.p.

[25] Rosser R, Kind P (1978) A scale of valuations of states of illness: is there a social consensus? International journal of epidemiology 7: 347–358. 744673 10.1093/ije/7.4.347

[26] Gross CP, Anderson GF, Powe NR (1999) The relation between funding by the National Institutes of Health and the burden of disease. N Engl J Med 340: 1881–1887. 10369852 10.1056/NEJM199906173402406

[27] World Bank (Several years) World development indicators. Washington, D.C.: World Bank. pp. v.

[28] Samb B, Evans T, Dybul M, Atun R, Moatti JP, et al (2009) An assessment of interactions between global health initiatives and country health systems. Lancet 373: 2137–2169. 19541040 10.1016/S0140-6736(09)60919-3

[29] Bendavid E, Miller G (2010) The US Global Health Initiative: informing policy with evidence. JAMA : the journal of the American Medical Association 304: 791–792. 20716743 10.1001/jama.2010.1189 PMC3816172

[30] Economist T (2011) Faster is not always better: Many children get the wrong kind of emergency care. The Economist: The Economist.

[31] Albain KS, Unger JM, Crowley JJ, Coltman CA Jr, Hershman DL (2009) Racial disparities in cancer survival among randomized clinical trials patients of the Southwest Oncology Group. J Natl Cancer Inst 101: 984–992. 19584328 10.1093/jnci/djp175 PMC2724852

[32] Chartier K, Caetano R (2010) Ethnicity and Health Disparities in Alcohol Research. Alcohol research & health : the journal of the National Institute on Alcohol Abuse and Alcoholism 33: 152–160. 21209793 PMC3887493

[33] Wallace TA, Martin DN, Ambs S (2011) Interactions among genes, tumor biology and the environment in cancer health disparities: examining the evidence on a national and global scale. Carcinogenesis 32: 1107–1121. 21464040 10.1093/carcin/bgr066 PMC3149201

[34] Grant WB, Peiris AN (2010) Possible role of serum 25-hydroxyvitamin D in black-white health disparities in the United States. Journal of the American Medical Directors Association 11: 617–628. 21029996 10.1016/j.jamda.2010.03.013

[35] Torgerson DG, Ampleford EJ, Chiu GY, Gauderman WJ, Gignoux CR, et al (2011) Meta-analysis of genome-wide association studies of asthma in ethnically diverse North American populations. Nature genetics 43: 887–892. 21804549 10.1038/ng.888 PMC3445408

[36] Bunker CH, Patrick AL, Konety BR, Dhir R, Brufsky AM, et al (2002) High prevalence of screening-detected prostate cancer among Afro-Caribbeans: the Tobago Prostate Cancer Survey. Cancer epidemiology, biomarkers & prevention : a publication of the American Association for Cancer Research, cosponsored by the American Society of Preventive Oncology 11: 726–729. 12163325

[37] Epstein S (2007) Inclusion : the politics of difference in medical research. Chicago: University of Chicago Press. ix, 413 p.p.

