# Supplementary material for: Attention to Local Health Burden and the Global Disparity of Health Research
Source: PLoS One. 2014 Apr 1;9(4):e90147. doi: 10.1371/journal.pone.0090147 (PMC3972174; doi:10.1371/journal.pone.0090147)
Supplement: File S1 — Supporting information, figures, and tables. Figure S1, 2004 global disability-adjusted life years (DALYs) and 2005 reviews, clinical trials and animal studies categorized by 19 broad WHO disease and disability categories. This correspondence the loose relationship between burden of disease and health knowledge (see Figure 1). Figure S2, Relationship between national disease burden and wealth. Scatterplots of national DALY rate (DALYs per 1000 people) and GNI per capita for each of 96 specific health conditions, where each point is a country. Also shown is the estimated influence (or regression slope) of logged DALY rate on logged GNI per capita, by condition, computed using ordinary least-squares (OLS) regression. Figure S3, Relationship between the national GDP per capita in 2004 and the quantity of reviews, clinical trials and animal studies published by researchers in 2005, by country, plotted on a logarithmic scale (to spread out countries for visual inspection). Each three character string corresponds to the unique ISO 3166-1 alpha-3 code associated with each country (see Figure 3 and Table S4 in File S1 for complete list). Table S1, Complete list of WHO Global Burden of Disease Categories. Table S2, Estimated Change in Global Number of Biomedical Articles with Changes in Global Health Burden (1990, 2004). Table S3, Estimated Change in Regional Number of Biomedical Articles with Changes in Regional Health Burden (1990, 2004). Table S4, Disease or Disease Category exacting the most DALYs. Table S5, Countries and their 3-Character Codes from Figure 3 . (DOCX) [file pone.0090147.s001.docx]

**Supporting Information for:**

**Attention to Local Health Burden**

**and the Global Disparity of Health Research**

Evans, J.A., Shim, J.M., Ioannidis, J.P.A.

**Materials and Methods**

*WHO diseases and the DALY measure.* Table S1 lists the complete collection of diseases considered in the WHO burden of disease project, including the residual categories that we excluded in our analysis because they made identification of comparable research articles impractical. Disability Adjusted Life years are defined as follows:

$$DALY=\left(deaths\times\varepsilon\right)+\left(cases\times\omega\times duration\right)$$

such that the number of deaths multiplied by ε—the standard life expectancy at age of death in years—is equivalent to years of life lost, and the number of incident cases multiplied by ω—a disease-specific disability weight—multiplied by the average duration in years of cases until remission or death is equivalent to years lost due to disability.[[1](#_ENREF_1)] The 1990 and 2004 studies discount the value of each subsequent year of life by 3% and then add an additional non-uniform age weight to give less value to years lived at young and older ages. The 2002 (and 2004) studies used 3% annual discounting and uniform age weighting.[[2](#_ENREF_2)]

*MEDLINE articles.* Disease relevant biomedical abstracts indexed in MEDLINE were used to calculate the amount of health knowledge relevant to global disorders. Articles indexed in MEDLINE, however, are all published in English and include only the most cited medically relevant journals. This inclusion criterion naturally censors historically important biomedical research published in other languages. Nevertheless, by 2003 and 2005, the core years at which articles were assessed for this analysis (524,728 and 583,843 articles, respectively), a large proportion of international biomedical research had come to be published primarily in English. As such, our analysis included 177,793 nonexclusive disease-article assignments in 2003 and 196,847 in 2005 (167,038 in 2003 and 191,830 in 2005 were in the country-level analysis—not all of the articles had country assignments). While it could have been reasonable to enlarge the time window in which articles were evaluated (e.g., 2-5 years following burden of disease assessments), we only had access to country-level data through the first quarter of 2006. As a result, we are unable to perform analysis with an expanded window of articles at the country level of analysis. While this limitation in the dependent variable inevitably influences the size of estimated coefficients in our models, it is unlikely to have influenced their direction or their comparative size across global and country-level models.

We initially assessed each article as having been produced by the country associated with each contributing institution. For example, a published international collaboration between U.S. and Burundi biomedical researchers would be credited to *both* countries. Because such a collaboration would be expected to draw from both collaborators, even if resources came from the U.S. and clinical access from Malawi (the poorest country in our database in 2004), this is a reasonable approach to measurement. We separately assessed article-country assignments based on only the richest of the contributing countries. This restricts the measure to indigenous research—the U.S.-Burundi collaboration would just be assigned to the U.S. and only a Burundi paper would be credited to Burundi. This alternative approach produced the same pattern of results.

In order to evaluate each link in the chain from GBD codes to articles, via ICD-9 codes and MeSH headings, we independently linked GBD and MEDLINE articles through several alternative approaches. We directly mapped strings from GBD terms into MEDLINE articles, from GBD-linked ICD-9 codes into MEDLINE articles, and from MeSH terms into MEDLINE articles. Differences in these alternate estimates of the number of global articles relevant to disease and disability yielded only small differences in the pattern of overall outcomes.

*Negative binomial models* (Tables 2A and 2B). These models predicted the number of articles published globally and indexed in MEDLINE relevant to one of 111 disorders, as a function of disability adjusted life-years (DALYs), market size, the prior stock of articles on the subject, and other controls, described below. Article numbers are discrete with high values concentrated in diseases that impose a large health toll on society or are considered biologically important, and varying widely among others. This behavior defies assumptions of the standard linear regression model, and so we estimate the relationship between citations and research strategies using a negative binomial model, a generalization of the Poisson model.

The Poisson distribution expresses the probability that a given number of events occur within a fixed interval if events occur with a known average rate and independently of the time since the prior event. In the Poisson distribution, the one parameter λ equals both mean and variance ();^[[1]](#footnote-1)^the tail of the distribution of articles is thick, however, with a higher variance than mean, and so we model it with the negative binomial distribution. The negative binomial distribution (*r*,*p*) represents the number of successes in a sequence of Bernoulli trials, each successful with probability *p*, before a specified (non-random) number *r* of failures occurs. It can also be viewed as a mixture of Poisson distributions, or a Poisson(λ) distribution, where λ is itself a random variable, distributed according to the gamma distribution . Like Poisson models [[3](#_ENREF_3)], negative binomial models assume that the logarithm of the expected value of the dependent variable can be modeled by a linear combination of unknown parameters. In this sense, it is similar to estimating a simple linear regression with the logarithm of citations as the dependent variable.

At the world-level analysis, we analyze 111 GBD codes at two time points (2002, 2004). Specifically, in order to test possible determinants of the production of biomedical knowledge at the global level, we analyze 111 GBD codes at two time points (2002 and 2004) and assume:

*Articles_td_ ~ NegativeBinomial(μ_td_)*

where *Articles_td_* are the number of articles published in year *t* about disease *d*. Then we estimate:

*Log(μ_td_) = α +β_1_DALY_td-1_ + β_2_market size_td-1_ + β_3_Articles_td-1_*  + *β_4_Disease category +* ε*_td_*

where *t* indicates year, *d* indicates each disease and the global market size is computed by *∑GNI per capita_k_ ×national DALY_k_* for each country *k*. We report the results from this cross-sectional time-series regression using a random effects (REM) modeling design because the data provide us only two time points, so close together that we did not believe that we could identify a causal effect. Nevertheless, a fixed effects (FEM) estimation that compares each disease only to itself over time (2002 and 2004) provides very similar estimates to those provided in Tables 2A and B, with the DALY a statistically insignificant and market size a significant predictor.

Here we also present global findings from the comparable analysis of 1990 and 2004, based on DALYs that incorporate non-uniform age weights. Table S2 presents the results of both random and fixed-effects negative binomial panel global models with same covariates as described in the article. Because this fourteen year span is so much larger than the two year span analyzed in the article, we were interested in not only comparing diseases to one another (random effects), but to themselves over time (fixed effects). All but one of these models suggests that the global burden of disease has no significant influence on the production of worldwide biomedical articles. The fixed-effect model controlling for disease and disability categories shows a weakly significant negative effect. The effect of the global market is larger in all of these models. For every 10 billion dollars of income lost to a disease globally, worldwide research increases by 10% to 15%.

In the country-level analysis, we use the 4,677 pairs of disease-country tuples at two time points with the total number of observations being 9,354.^[[2]](#footnote-2)^ In parallel with the global analysis, we specify the association of national or “local” DALY on the production of biomedical articles, controlling for global DALY and historical articles on those disorders. Specifically, we estimate the following country-level model:

*Log(Articles_tkd_ ) = α +β_1_National DALY_tkd-1_ + β_2_Global DALY_td-1_+β_3_National articles_tkd-1_ + β_4_Global articles_td-1_ + β_5_Broad disease category +*ε*_dkt_*

where *t* represents year, *k* country and *d* disease. As in the global analysis, we report results from cross-sectional time-series regressions under a random effects (REM) modeling specification. A fixed effects specification limits the number of countries allowable in the analysis, because several only received a DALY score for one year. Moreover, fixed effects models could not be estimated for the models with broad disease category indicator variables. A fixed effects specification of the model with only DALY as a predictor produces very different estimates, including a negative relationship between national DALY and national disease-specific articles (an increase in a million national DALYs corresponds to a decrease in articles by about 50%). This is likely a result of the lag between DALY and articles. For example, when the lag was increased to 2 years, the decrease dropped to 5%, but our country-tagged article data only go through the first quarter of 2006, as described earlier and so a 3-year lag specification was not possible. As a result, we interpret our estimated effect as an association and not a causal influence.

We also estimated models similar to those just described, but at the regional level of analysis to take advantage of shifts between 1990 and 2004. In 1990, the WHO estimated DALYs for diseases in the following regions: Established Market Economies; Former Socialist Europe; India; China; Other Asia and Islands; Sub-Saharan Africa; Latin America; and the Middle East. These “regions” convolute geography, political and economic development, but because the countries assigned to each region were clear, we could reevaluate them again in 2004. When we estimate the country-level models at the region level of analysis for 1990 and 2004, the coefficients do not demonstrate the same strong, consistent influence of regional DALYs on the regional publication of disease-relevant articles as in the country case (see Table S3). Nevertheless, the effect was stronger than in the global model. When regional DALYs were regressed alone on the log number of articles with a random effects specification, for every 10 million additional regional DALYs lost to a disease, there appears to be a 21% increase in regional articles researching the topic. When global DALYs are added to the model, the regional DALYs become insignificant. When cumulative global articles are added to the model, only these remain significant predictors of future regional articles. Thus, the regional models suggest a larger influence of disease burden than the global models, but a smaller one than the country models described in the article.

*Alternative modeling specifications.* We attempted alternative specifications of several measurements in the model to assess the model’s sensitivity. For example, we associated each article to only richest or poorest country associated with an author, rather than all countries hosting an author. We also added an indicator variable to identify the year of case. These alternate specifications did not change the pattern of results.

**National Wealth and Disease**

Figures 2 and A2 illustrate the global inequality of diseases and disease categories by plotting the relationship between disease DALY rate (DALYs lost to the disease per 1000 people) for each country and that country’s gross domestic product (GDP) per capita. Both figures also list the estimated effect of logged GDP per capita on logged DALY rate, which equals the slope of the line that best fits the plotted points. More negative slopes suggest that the disease or disease category is distinctive to poor countries, and more positive slopes suggest that they are distinctive to rich countries. Figure S1 shows how malaria, tetanus, chagas disease, measles, Vitamin A deficiency, lymphatic filariasis, schistosomiasis, and diphtheria are the conditions most characteristic of poor countries. Cancers and Alzheimer’s disease—conditions most likely to afflict the old—are most characteristic of rich countries.

Table S4 shows the top-level, coarse-grained and fine-grained disease categories that exact the most DALYs for each GDP decile of the world. Deciles 1-3 (e.g., Ethiopia, Nigeria, India) are most afflicted with communicable, infectious and parasitic conditions including lower respiratory infections and HIV/AIDS. Higher GDP deciles (e.g., China, Iran, Russia, Germany, the United States) are most burdened by neuro-psychiatric conditions, especially unipolar depression. Note that the conditions exacting the most DALYs in richer countries cost many fewer than those that exact the most in poor countries. This suggests that poor countries have worse health overall, but also that ill health in rich countries is more evenly distributed across many disorders than ill health in poor countries.

Table S5 lists the 3 character country codes used in Figure 3. Although rich countries typically spend more on research, there are exceptions. Small, rich countries like Luxemborg (LUX) and Qatar (QAT) fund little, but benefit from relevant health research performed by their neighbors and trade partners. By contrast, large, relatively poor countries like India (IND) and China (CHN) fund more than would be expected likely because they can reap large-scale benefits from even moderate investments in health knowledge.

| Table S1. Complete list of WHO Global Burden of Disease Categories | | |
| --- | --- | --- |
| Broad categories | **Detailed categories (in order of most DALYs)^†^** |  |
| Group I. Communicable, maternal, peri-natal and nutritional conditions | |  |
| Infectious or parasitic diseases | Diarrheal diseases, HIV/AIDS, Tuberculosis, Malaria, Measles, Menigitis, Pertussis, Lymphatic filarisasis, Tetanus, Gohorrhoea, Syphilis, Hepatitis B, Leishmaniasis, Ascariasis, Schistosomiasis, Trypanosomiasis, Trachoma, Hookworm disease, Trichuriasis, Japanese encephalitis, Dengue, Chagas disease, Onchocerciasis, Leprosy, Diphtheria, Poliomyelitis, Other STDs*, Hepatitis C*, Other intestinal infections*, Other infectious diseases* |  |
| Respiratory infections | Lower respiratory infections, Upper respiratory infections, Otitis media |  |
| Maternal conditions | Abortion, Maternal sepsis, Maternal haemorrhage, Hypertensive disorders of pregnancy, Ostructed labor*, Other maternal conditions* |  |
| Peri-natal conditions | Low birth weight, Birth asphyxia and birth trauma, Other perinatal conditions* |  |
| Nutritional deficiencies | Protein-energy malnutrition, Iron-deficiency anaemia, Lodine deficiency, Vitamin A deficiency, Other nutritional disorders* |  |
| Group II. Non-communicable conditions | |  |
| Malignant neoplasms | Trachea/Bronchus/Lung cancers, Stomach cancer, Liver cancer, Breast cancer, Colon and rectum cancers, Leukaemia, Oesophagus cancer, Lymphomas and multiple myeloma, Mouth and oropharynx cancers, Cervix uteri cancer, Pancreas cancer, Prostate cancer, Ovary cancer, Bladder cancer, Corpus uteri cancer, Melanoma and other skin cancers, Other malignant neoplasms*, Other neoplasms* |  |
| Endocrine disorders | Diabetes mellitus, Endocrine disorders |  |
| Neuro-psychiatric conditions | Unipolar depressive disorders, Alcohol use disorders, Schizophrenia, Bipolar affective disorder, Alzheimer and other dementias, Mental retardation from lead exposure, Drug use disorders, Epilepsy, Migraine, Panic disorder, Obsessive-compulsive disorder, Insomnia, Post-traumatic stress disorder, Parkinson disease, Multiple sclerosis, Other neuro-psychiatric disorders* |  |
| Sense organ diseases | Vision disorders (age-related), Hearing loss (adult onset), Cataracts, Glaucoma, Other sense organ disorders* |  |
| Cardiovascular diseases | Ischaemic heart disease, Cerebrovascular disease, Hypertensive heart disease, Inflammatory heart disease, Rheumatic heart disease, Other cardiovascular diseases* |  |
| Respiratory diseases | Chronic obstructive pulmonary disease, Asthma, Other respiratory diseases* |  |
| Digestive diseases | Cirrhosis of the liver, Peptic ulcer disease, Appendicitis, Other digestive diseases* |  |
| Genito-urinary diseases | Nephritis and nephrosis, Benign prostatic hypertrophy, Other genito-urinary system diseases* |  |
| Skin diseases | Skin diseases |  |
| Musculoskeletal diseases | Osteoarthritis, Rheumatoid arthritis, Gout, Low back pain, Other muscularskeletal diseases* |  |
| Congenital anomalies | Congenital heart anomalies, Down syndrome, Spina bifida, Anencephaly, Cleft palate, Abdominal wall defect, Cleft lip, Oesophageal atresia, Anorectal atresia*, Renal agenesis*, Other congenital anomalies* |  |
| Oral conditions | Dental caries, Edentulism, Periodontal disease, Other oral diseases* |  |
| Group III. Injuries | |  |
| Unintentional injuries | Road traffic accidents, Falls, Fires, Drownings, Poisonings, Other unintentional injuries* |  |
| Intentional injuries | Violence, Self-inflicted injuries, War*, Other intentional injuries* |  |
| * Residual categories excluded from our analyses. | | |

| Table S2. Estimated Change in Global Number of Biomedical Articles with Changes in Global Health Burden (1990, 2004) ^a^ | | | | | | | | |
| --- | --- | --- | --- | --- | --- | --- | --- | --- |
|  | Model 1 | | Model 2 | | Model 3 | | Model 4^†^ | |
|  | % change | 95% C.I.^‡^ | % change | 95% C.I. | % change | 95% C.I. | % change | 95% C.I. |
| Random effects models |  |  |  |  |  |  |  |  |
| Global DALYs  (10 millions) | 4.9 | -1.8 – 12.0 | 0.0 | -5.8 – 6.1 | -2.6 | -8.6 – 3.6 | -1.1 | -7.8 – 6.1 |
| Market size  ($10 billions) |  |  | 11.2^**^ | 8.6 – 13.8 | 11.3^**^ | 8.8 – 13.9 | 10.4^**^ | 7.5 – 13.4 |
| Cumulative Global  articles (10 thousands) |  |  |  |  | 2.7^**^ | 2.0 – 3.4 | 2.7^**^ | 2.0 –3.4 |
| Fixed effects models |  |  |  |  |  |  |  |  |
| Global DALYs  (10 millions) | -.7 | -8.7 – 8.1 | -2.0 | -7.8 – 4.1 | -4.8 | -10.7 – 1.5 | -7.2^*^ | -12.8 – -1.3 |
| Market size  ($10 billions) |  |  | 13.8^**^ | 10.7 – 16.9 | 13.7^**^ | 10.7 – 16.8 | 14.7^**^ | 11.7 – 17.9 |
| Cumulative Global  articles (10 thousands) |  |  |  |  | 2.3^**^ | 1.6 – 3.1 | 2.4^**^ | 1.7 –3.2 |
| ^a^ This supplementary analysis shows that the patterns in Table 2A remain unchanged even over a wider time period.  Each model contains 200 cases (100 diseases in 1990 and 2004).  ^†^ Model 4 controls for the 19 broad disease / disability categories from Table S1  ^‡^ 95% confidence interval  ^*^ *p* < .05; ^**^ *p* < .01; | | | | | | | | |

| Table S3. Estimated Change in Regional Number of Biomedical Articles with Changes in Regional Health Burden (1990, 2004) ^a^ | | | | | | | | | | |
| --- | --- | --- | --- | --- | --- | --- | --- | --- | --- | --- |
|  | Model 1 | | Model 2 | | Model 3 | | Model 4 | | Model 5^†^ | |
|  | % Δ | 95% C.I.^‡^ | % Δ | 95% C.I. | % Δ | 95% C.I. | % Δ | 95% C.I. | % Δ | 95% C.I. |
| Random effects models |  |  |  |  |  |  |  |  |  |  |
| Regional DALYs  (10 millions) | 21.1^*^ | 0.1 – 46.7 | -18.4 | -40.5 – 9.9 | 21.0^**^ | 0.1 – 46.4 | -12.2 | -33.9 – 16.4 | -12.8 | -34.3 – 15.7 |
| Global DALYs  (10 millions) |  |  | 10.1^**^ | 5.0 – 15.5 |  |  | 8.4^**^ | 3.5 – 13.7 | 9.5^**^ | 4.4 – 14.8 |
| Cumulative Regional  articles (10 thousands) |  |  |  |  | 9.7^**^ | 7.6 – 11.8 | -.2 | -2.4 – 2.1 | 0.0 | -2,3 – 2.2 |
| Cumulative Global  articles (10 thousands) |  |  |  |  |  |  | 8.4^**^ | 7.5 – 9.3 | 8.3^**^ | 7.4 – 9.2 |
| Fixed effects models |  |  |  |  |  |  |  |  |  |  |
| Regional DALYs  (10 millions) | 2.6 | -23.1 – 36.9 | -20.7 | -47.7 – 20.1 | 2.3 | -23.3 – 36.6 | -13.4 | -42.4 – 30.1 | -10.9 | -41.1 – 34.8 |
| Global DALYs  (10 millions) |  |  | 6.1^+^ | -.6 – 13.2 |  |  | 4.0 | -2.6 – 11.0 | 0.2 | -6.2 – 7.1 |
| Cumulative Regional  articles (10 thousands) |  |  |  |  | 8.1^**^ | 5.4 – 11.0 | 0.9 | -3.9 – 2.1 | -1.0 | -4.0 –2.1 |
| Cumulative Global  articles (10 thousands) |  |  |  |  |  |  | 7.6^**^ | 6.5 – 8.7 | 7.9^**^ | 6.8 –9.1 |
| ^a^ This supplementary analysis shows that the patterns in Table 2B remain largely unchanged at the regional level over a wider time period.  Each model contains 1374 cases (100 diseases and 8 regions in 1990 and 2004). We used the WHO’s 1990 regional classification where the  eight regions are Established Market Economies (EME), Formerly Socialist Economies of Europe (FSE), China (CHI), India (IND), Other Asia  and Islands (OAI), Latin America and the Caribbean (LAC), Middle East Crescent (MEC), and Sub-Saharan Africa (SSA).  ^†^ Model 2 controls for the 3 top level disease / disability categories from Table S1. With the 19 coarse disease categories included in prior models,  these models could not be estimated.  ^‡^ 95% confidence interval  ^+^ *p* < .10; ^*^ *p* < .05; ^**^ *p* < .01; | | | | | | | | | | |

| Table S4. Disease or Disease Category exacting the most DALYs  (DALYs in million years in parentheses) | | | | |
| --- | --- | --- | --- | --- |
| Decile GDP per capita | Highest GDP  in decile | Top level category | Coarse category (of 19) | Disease |
| 1 | Ethiopia | Communicable, maternal, perinatal, nutritional (94.7) | Infectious parasitic (55.2) | Lower respiratory infections (22.1) |
| 2 | Nigeria | Communicable, maternal, perinatal, nutritional (118.6) | Infectious parasitic (77.2) | HIV/AIDS (20.5) |
| 3 | India | Communicable, maternal, perinatal, nutritional (161.3) | Infectious parasitic (79.6) | Lower respiratory infections (32.3) |
| 4 | China | Noncommunicable (164.5) | Neuro-psychiatric conditions (45.4) | Cerebrovascular disease (16.3) |
| 5 | Iran | Noncommunicable (25.4) | Neuro-psychiatric conditions (7.8) | Ischaemic heart disease (3.6) |
| 6 | Russia | Noncommunicable (46.0) | Infectious parasitic (16.4) | HIV/AIDS (11.5) |
| 7 | Brazil | Noncommunicable (37.4) | Neuro-psychiatric conditions (12.6) | Unipolar depressive disorders (4.8) |
| 8 | Mexico | Noncommunicable (16.8) | Neuro-psychiatric conditions (5.4) | Unipolar depressive disorders (1.8) |
| 9 | Germany | Noncommunicable (31.2) | Neuro-psychiatric conditions (10.3) | Unipolar depressive disorders (3.3) |
| 10 | United States | Noncommunicable (51.6) | Neuro-psychiatric conditions (18.3) | Unipolar depressive disorders (6.4) |

| Table S5. Countries and their 3-Character Codes from Figure 3 | | | | | | | |
| --- | --- | --- | --- | --- | --- | --- | --- |
| ABW | Aruba | DNK | Denmark | LAO | Lao People's Democratic Rep. | QAT | Qatar |
| AFG | Afghanistan | DOM | Dominican Republic | LBN | Lebanon | REU | Réunion |
| AGO | Angola | DZA | Algeria | LBR | Liberia | ROU | Romania |
| AIA | Anguilla | ECU | Ecuador | LBY | Libyan Arab Jamahiriya | RUS | Russian Federation |
| ALA | Åland Islands | EGY | Egypt | LCA | Saint Lucia | RWA | Rwanda |
| ALB | Albania | ERI | Eritrea | LIE | Liechtenstein | SAU | Saudi Arabia |
| AND | Andorra | ESH | Western Sahara | LKA | Sri Lanka | SDN | Sudan |
| ARE | United Arab Emirates | ESP | Spain | LSO | Lesotho | SEN | Senegal |
| ARG | Argentina | EST | Estonia | LTU | Lithuania | SGP | Singapore |
| ARM | Armenia | ETH | Ethiopia | LUX | Luxembourg | SGS | South Georgia |
| ASM | American Samoa | FIN | Finland | LVA | Latvia | SHN | St Helena, Ascension, Tristan |
| ATA | Antarctica | FJI | Fiji | MAC | Macao | SJM | Svalbard and Jan Mayen |
| ATF | French S. Terr. | FLK | Falkland Islands | MAF | Saint Martin (French part) | SLB | Solomon Islands |
| ATG | Antigua and Barbuda | FRA | France | MAR | Morocco | SLE | Sierra Leone |
| AUS | Australia | FRO | Faroe Islands | MCO | Monaco | SLV | El Salvador |
| AUT | Austria | FSM | Micronesia | MDA | Moldova, Republic of | SMR | San Marino |
| AZE | Azerbaijan | GAB | Gabon | MDG | Madagascar | SOM | Somalia |
| BDI | Burundi | GBR | United Kingdom | MDV | Maldives | SPM | Saint Pierre & Miquelon |
| BEL | Belgium | GEO | Georgia | MEX | Mexico | SRB | Serbia |
| BEN | Benin | GGY | Guernsey | MHL | Marshall Islands | STP | Sao Tome and Principe |
| BES | Bonaire, S. Eusta., Saba | GHA | Ghana | MKD | Macedonia | SUR | Suriname |
| BFA | Burkina Faso | GIB | Gibraltar | MLI | Mali | SVK | Slovakia |
| BGD | Bangladesh | GIN | Guinea | MLT | Malta | SVN | Slovenia |
| BGR | Bulgaria | GLP | Guadeloupe | MMR | Myanmar | SWE | Sweden |
| BHR | Bahrain | GMB | Gambia | MNE | Montenegro | SWZ | Swaziland |
| BHS | Bahamas | GNB | Guinea-Bissau | MNG | Mongolia | SXM | Sint Maarten (Dutch) |
| BIH | Bosnia and Herz. | GNQ | Equatorial Guinea | MNP | Northern Mariana Islands | SYC | Seychelles |
| BLM | Saint Barthélemy | GRC | Greece | MOZ | Mozambique | SYR | Syrian Arab Republic |
| BLR | Belarus | GRD | Grenada | MRT | Mauritania | TCA | Turks and Caicos Islands |
| BLZ | Belize | GRL | Greenland | MSR | Montserrat | TCD | Chad |
| BMU | Bermuda | GTM | Guatemala | MTQ | Martinique | TGO | Togo |
| BOL | Bolivia | GUF | French Guiana | MUS | Mauritius | THA | Thailand |
| BRA | Brazil | GUM | Guam | MWI | Malawi | TJK | Tajikistan |
| BRB | Barbados | GUY | Guyana | MYS | Malaysia | TKL | Tokelau |
| BRN | Brunei Darussalam | HKG | Hong Kong | MYT | Mayotte | TKM | Turkmenistan |
| BTN | Bhutan | HMD | Heard Island & McDonald Isles | NAM | Namibia | TLS | Timor-Leste |
| BVT | Bouvet Island | HND | Honduras | NCL | New Caledonia | TON | Tonga |
| BWA | Botswana | HRV | Croatia | NER | Niger | TTO | Trinidad and Tobago |
| CAF | Central African Republic | HTI | Haiti | NFK | Norfolk Island | TUN | Tunisia |
| CAN | Canada | HUN | Hungary | NGA | Nigeria | TUR | Turkey |
| CCK | Cocos (Keeling) Islands | IDN | Indonesia | NIC | Nicaragua | TUV | Tuvalu |
| CHE | Switzerland | IMN | Isle of Man | NIU | Niue | TWN | Taiwan |
| CHL | Chile | IND | India | NLD | Netherlands | TZA | Tanzania |
| CHN | China | IOT | British Indian Ocean Territories | NOR | Norway | UGA | Uganda |
| CIV | Côte d'Ivoire | IRL | Ireland | NPL | Nepal | UKR | Ukraine |
| CMR | Cameroon | IRN | Iran, Islamic Republic of | NRU | Nauru | UMI | US Minor Outlying Islands |
| COD | Congo, the DRC | IRQ | Iraq | NZL | New Zealand | URY | Uruguay |
| COG | Congo | ISL | Iceland | OMN | Oman | USA | United States |
| COK | Cook Islands | ISR | Israel | PAK | Pakistan | UZB | Uzbekistan |
| COL | Colombia | ITA | Italy | PAN | Panama | VAT | Holy See (Vatican City) |
| COM | Comoros | JAM | Jamaica | PCN | Pitcairn | VCT | St. Vincent & Grenadines |
| CPV | Cape Verde | JEY | Jersey | PER | Peru | VEN | Venezuela |
| CRI | Costa Rica | JOR | Jordan | PHL | Philippines | VGB | Virgin Islands, British |
| CUB | Cuba | JPN | Japan | PLW | Palau | VIR | Virgin Islands, U.S. |
| CUW | Curaçao | KAZ | Kazakhstan | PNG | Papua New Guinea | VNM | Viet Nam |
| CXR | Christmas Island | KEN | Kenya | POL | Poland | VUT | Vanuatu |
| CYM | Cayman Islands | KGZ | Kyrgyzstan | PRI | Puerto Rico | WLF | Wallis and Futuna |
| CYP | Cyprus | KHM | Cambodia | PRK | Korea, Dem. Peoples Rep. | WSM | Samoa |
| CZE | Czech Republic | KIR | Kiribati | PRT | Portugal | YEM | Yemen |
| DEU | Germany | KNA | Saint Kitts and Nevis | PRY | Paraguay | ZAF | South Africa |
| DJI | Djibouti | KOR | Korea | PSE | Palestinian Territory | ZMB | Zambia |
| DMA | Dominica | KWT | Kuwait | PYF | French Polynesia | ZWE | Zimbabwe |

Figure S1. 2004 global disability-adjusted life years (DALYs) and 2005 reviews, clinical trials and animal studies categorized by 19 broad WHO disease and disability categories. This correspondence the loose relationship between burden of disease and health knowledge (see Figure 1).

Figure S2. Relationship between national disease burden and wealth. Scatterplots of national DALY rate (DALYs per 1000 people) and GNI per capita for each of 96 specific health conditions, where each point is a country. Also shown is the estimated influence (or regression slope) of logged DALY rate on logged GNI per capita, by condition, computed using ordinary least-squares (OLS) regression.

Figure S3. Relationship between the national GDP per capita in 2004 and the quantity of reviews, clinical trials and animal studies published by researchers in 2005, by country, plotted on a logarithmic scale (to spread out countries for visual inspection). Each three character string corresponds to the unique ISO 3166-1 alpha-3 code associated with each country (see Figure 3 and Table S4 for complete list).

1. Organization WH (2011) Metrics: Disability-Adjusted Life Year (DALY). Health statistics and health information systems.

2. Organization WH (2011) Disability weights, discounting and age weighting of DALYs. Health statistics and health information systems.

3. Hausman J, Hall BH, Griliches Z (1984) Econometric-Models for Count Data with an Application to the Patents R and D Relationship. Econometrica 52: 909-938.

1. The Poisson probability mass function is$\frac{\lambda^{k}e^{-\lambda}}{k!}$, where *k* is the number of occurrences of the event in question, and λ is the expected number of occurrences in a given interval. [↑](#footnote-ref-1)
2. In theory, the maximum number of cases – i.e. GBD codes-country pairs – is 21,312 (= 111 codes × 192 countries). However, cases that are missing a value in any of the variables in the regression equations have been excluded from our analyses. [↑](#footnote-ref-2)
